# Molecular architectures of benzoic acid-specific type III polyketide synthases

**DOI:** 10.1107/S2059798317016618

**Published:** 2017-11-30

**Authors:** Charles Stewart, Kate Woods, Greg Macias, Andrew C. Allan, Roger P. Hellens, Joseph P. Noel

**Affiliations:** a Howard Hughes Medical Institute, The Salk Institute for Biological Studies, La Jolla, CA 92037, USA; b Macromolecular X-ray Crystallography Facility, Office of Biotechnology, Iowa State University, 0202 Molecular Biology Building, 2437 Pammel Drive, Ames, IA 50011, USA; c The New Zealand Institute for Plant and Food Research Limited (PFR), Auckland, New Zealand; d School of Biological Sciences, University of Auckland, Auckland, New Zealand; e Queensland University of Technology, Brisbane, Queensland 4001, Australia

**Keywords:** chalcone synthase, biphenyl synthase, benzophenone synthase, polyketide synthase, thiolase, benzoyl-CoA

## Abstract

Biphenyl synthase and benzophenone synthase constitute an evolutionarily distinct clade of type III polyketide synthases (PKSs) that use benzoic acid-derived substrates to produce defense metabolites in plants. The use of benzoyl-CoA as an endogenous substrate is unusual for type III PKSs. Moreover, sequence analyses indicate that the residues responsible for the functional diversification of type III PKSs are mutated in benzoic acid-specific type III PKSs. In order to gain a better understanding of structure–function relationships within the type III PKS family, the crystal structures of biphenyl synthase from *Malus* × *domestica* and benzophenone synthase from *Hypericum androsaemum* were compared with the structure of an archetypal type III PKS: chalcone synthase from *Malus* × *domestica*. Both biphenyl synthase and benzophenone synthase contain mutations that reshape their active-site cavities to prevent the binding of 4-coumaroyl-CoA and to favor the binding of small hydrophobic substrates. The active-site cavities of biphenyl synthase and benzophenone synthase also contain a novel pocket associated with their chain-elongation and cyclization reactions. Collectively, these results illuminate structural determinants of benzoic acid-specific type III PKSs and expand the understanding of the evolution of specialized metabolic pathways in plants.

## Abstract 2

The structures of biphenyl synthase, of biphenyl synthase complexed with benzoyl-CoA and of benzophenone synthase are compared with that of a chalcone synthase homolog. These structures reveal that benzoic acid-specific type III polyketide synthases contain distinct structural elements, including a novel pocket, which underlie their evolutionary emergence.

## 1. Introduction   

**Figure 1:**
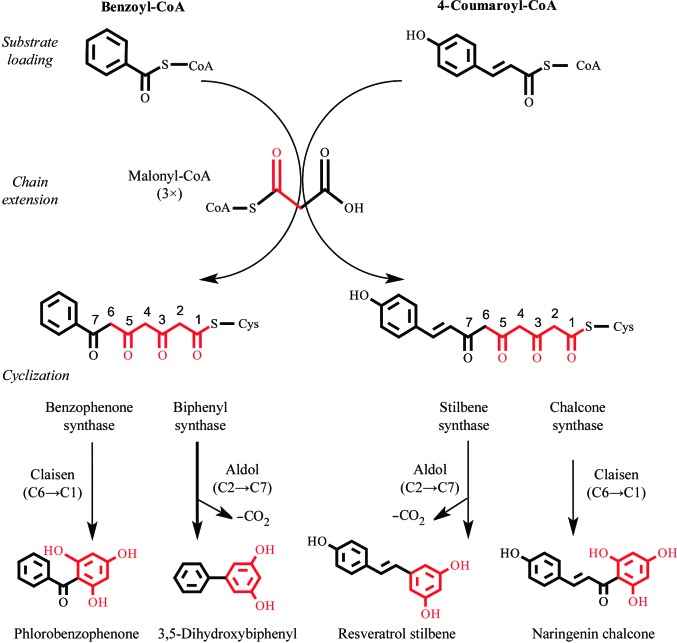
Biochemical reactions of benzophenone synthase and biphenyl synthase compared with the type III PKS archetypes chalcone synthase and stilbene synthase. Acetyl units derived from the decarboxylative condensation of malonyl-CoA are shown in red. Only dominant products and reactions known to occur *in planta* are shown.

Benzoic acid-specific type III polyketide synthases (PKSs) constitute a distinct clade within the type III PKS family that use benzoic acid-derived substrates (for example benzoyl-CoA, 3-hydroxybenzoyl-CoA and salicoyl-CoA) to produce phytoalexins and pharmacologically active compounds (Beerhues & Liu, 2009 ▸; Fig. 1 ▸). Biphenyl synthase (BIS) generates the core chemical scaffolds of biphenyl and dibenzofuran phytoalexins commonly found in the Pyrinae subtribe (Rosaceae; Liu *et al.*, 2007 ▸; Khalil *et al.*, 2013 ▸). The Pyrinae contain several economically important species, including apple (*Malus* × *domestica*), pear (*Pyrus communis*) and mountain ash (*Sorbus aucuparia*). Apples increased their expression of BIS after inoculation with the fireblight bacterium *Erwinia amylovora* (Chizzali *et al.*, 2011 ▸). Furthermore, BIS transcripts as well as biphenyl and dibenzofuran compounds have been isolated from the transition zone between necrotic and healthy tissues in both apples and pears after inoculation with the fireblight bacterium (Chizzali *et al.*, 2012 ▸, 2016 ▸). Additionally, when challenged with *Venturia inaequalis*, the causative fungus of apple scab, cell cultures of *S. aucuparis* and a scab-resistant *M. domestica* cultivar produced biphenyl and dibenzofuran metabolites (Hüttner *et al.*, 2010 ▸; Khalil *et al.*, 2013 ▸; Hrazdina & Borejsza-Wysocki, 2003 ▸). Additionally, the promiscuous *in vitro* activity of BIS with salicoyl-CoA was exploited to develop an artificial metabolic system in *Escherichia coli* that is capable of producing 4-hydroxycoumarin, an immediate precursor to synthetic anticoagulants (for example warfarin; Liu *et al.*, 2010 ▸; Lin *et al.*, 2013 ▸). In contrast to BIS, benzophenone synthase (BPS) generates the core chemical scaffolds of xanthones, guttiferones and sampsoniones that are prominently found in the closely related Hypericaceae and Clusiaceae families (Liu *et al.*, 2003 ▸; Nualkaew *et al.*, 2012 ▸). Xanthones are associated with diverse biological functions in *Hypericum* spp., including antimicrobials, UV pigments and antioxidants (Gronquist *et al.*, 2001 ▸). Cell cultures of *H. calycinum* produced xanthones in response to yeast elicitation (Gaid *et al.*, 2012 ▸). Similarly, elicitation of *H. perforatum* cell cultures with *Agrobacterium tumefaciens* led to an increase in BPS transcripts and xanthone accumulation (Franklin *et al.*, 2009 ▸). Lastly, polyisoprenylated benzophenone derivatives are pharmacologically active and have served as lead compounds for drug development (Acuña *et al.*, 2009 ▸; Wang *et al.*, 2016 ▸).

Type III PKS catalysis involves the loading of a CoA-tethered substrate onto an active-site cysteine followed by chain elongation *via* iterative decarboxylative Claisen condensations of malonyl-CoA. Linear poly­ketide intermediates undergo a terminal intramolecular cyclization reaction to produce the final cyclic polyketide product (Austin & Noel, 2003 ▸). Changes in steric effects and electrostatic inter­actions within the active-site cavities of type III PKSs underlie biochemical variations in substrate preference, chain-elongation and cyclization patterns during catalysis (Jez, Austin *et al.*, 2000 ▸; Austin, Izumikawa *et al.*, 2004 ▸; Austin, Bowman *et al.*, 2004 ▸). The reactions catalyzed by benzoic acid-specific type III PKSs are biochemically and evolutionarily distinct from those catalyzed by the type III PKS archetypes chalcone synthase (CHS) and stilbene synthase (STS). Firstly, as stated above, the substrate preferences of BIS and BPS for benzoyl-CoA are unusual in the type III PKS family. No other type III PKSs are known to use benzoyl-CoA *in planta*. Secondly, sequence comparisons indicate that the molecular determinants of cyclization specificity in BIS and BPS are not conserved in CHSs and STSs (Liu *et al.*, 2007 ▸). Thirdly, molecular phylogenetic analyses indicate that BIS and BPS form a distinct clade within the type III PKS family, separate from CHS homologs from the same species and other functionally diverse type III PKSs (Liu *et al.*, 2007 ▸). This evolutionary pattern differs from the relationship of STSs to CHSs (Tropf *et al.*, 1994 ▸).

The lack of structural data for benzoic acid-specific type III PKSs limits our understanding of the enzymology of type III PKSs as well as the evolution of associated metabolic pathways. Our goal for this study was to identify structural idiosyncrasies of benzoic acid-specific type III PKSs *via* comparative analyses of the crystal structures of CHS, BIS and BPS.

## 2. Methods   

### 2.1. Chemicals   

Benzoyl-CoA, malonyl-CoA, naringenin and 4-hydroxy­coumarin were all purchased from Sigma (USA); 4-coumaroyl-CoA was purchased from TransMIT (Germany). 3,5-Dihydroxybi­phenyl was prepared as described previously by Nilsson & Norin (1963 ▸) and was purified with a Waters preparative LCMS FractionLynx system using mass and UV-directed fractionation on an XBridge Prep OBD C18 5 µm 30 × 100 mm column, with a gradient from 5:95:0.1(*v*:*v*:*v*) water:acetonitrile:formic acid to acetonitrile (0.1% formic acid) over 8 min with a flow rate of 30 ml min^−1^. Its identity was confirmed by comparison of its ^1^H NMR spectrum in MeOD-d_4_ and *m*/*z* values with those reported in the literature (Liu *et al.*, 2004 ▸). Salicoyl-CoA (2-hydroxybenzoyl-CoA) was prepared as described elsewhere (Sidenius *et al.*, 2004 ▸) and was purified *via* preparative LCMS as described above. The identity of salicoyl-CoA was confirmed by comparison of its ^1^H NMR spectrum in MeOD-d_4_ and *m*/*z* values with those reported in the literature (Guo *et al.*, 2009 ▸).

### 2.2. Cloning and recombinant protein expression and purification   

cDNAs for *MdCHS2*, *MdBIS1* and *MdBIS3* were derived from a cDNA library of *M. domestica* and inserted into the pBluescript SK+ vector in the laboratory of A. C. Allan and R. P. Hellens at The New Zealand Institute for Plant and Food Research Ltd (Newcomb *et al.*, 2006 ▸). For recombinant expression, the *MdCHS2*, *MdBIS1* and *MdBIS3* cDNAs were inserted between the NcoI and EcoRI restriction sites of the expression vector pHIS8 which, under the control of a T7 promoter, yields target proteins fused to an N-terminal octahistidine tag (Jez, Ferrer *et al.*, 2000 ▸). Subcloning occurred as follows. Firstly, NcoI digestion sites were added to each pBluescript template using the QuikChange (Agilent) PCR method following the manufacturer’s protocol. The primers used were MdCHS2-forward, 5′-CTCGACGGTCACC**ATG**GTTTTTATATCCGATCGTCGAGAAAGATC-3′; MdCHS2-reverse, 5′-GATCTTTCTCGACGATCGGATATAAAAAC**CAT**GGTGACCGTCGAG-3′; MdBIS1-forward, 5′-TAACCAAAGGCGCC**ATG**GGGCAAGTTGAAGAGC-3′; MdBIS1-reverse, 5′-GCTCTTCAACTTGCCCC**ATG**GCGCCTTTGGTTA-3′; MdBIS3-forward, 5′-CTCATTCTTAACCAAAGGCGCC**ATG**GAGCTAGTTAAAGAGCAGATATAAA-3′; MdBIS3-reverse, 5′-TTTATATCTGCTCTTTAACTAGCTC**CAT**GGCGCCTTTGGTTAAGAATGAG-3′ (NcoI sites are underlined and the translation start sites are shown in bold). Plasmid preparations (Qiagen) of NcoI-mutated pBluescript constructs were digested with NcoI and EcoRI. The approximately 1.2 kb digestion fragments were gel-purified and ligated with NcoI/EcoRI-digested pHIS8. The correct reading frames for the pHIS8 constructs were obtained using the QuikChange PCR method and the following sets of primers: MdCHS2-forward, 5′-TCCGCGTGGTTCC**ATG**GTGACCGTCG-3′; MdCHS2-reverse, 5′-CGACGGTCACC**ATG**GAACCACGCGGA-3′; MdBIS1 and MdBIS3-forward, 5′-TCCGCGTGGTTCC**ATG**GCGCCTTTGG-3′; MdBIS1 and MdBIS3-reverse, 5′-CCAAAGGCGCC**ATG**GAACCACGCGGA-3′ (NcoI sites are underlined and the translation start sites are shown in bold). All primers were purchased from Integrated DNA Technologies. The fidelity of all constructs was confirmed by sequencing (Eton Biosciences, USA).

Expression constructs were heterologously overexpressed in *E. coli* BL21 (DE3) (Novagen) expression hosts. *E. coli* cultures were grown at 37°C in TB medium (Invitrogen) to an optical density (600 nm) of 1.0, induced with 0.5 m*M* isopropyl β-d-thiogalactopyranoside and allowed to grow overnight at 22°C. Bacterial cells were harvested by centrifugation, resuspended in lysis buffer [50 m*M* Tris–HCl pH 8.0, 500 m*M* NaCl, 20 m*M* imidazole, 1%(*v*/*v*) Tween 20, 10%(*v*/*v*) glycerol, 10 m*M* β-mercaptoethanol] and lysed by sonication. Recombinant proteins were isolated from the *E. coli* lysate by affinity chromatography with nickel–nitrilotriacetic acid-coupled agarose (Qiagen) and eluted with buffer (lysis buffer without detergent) containing 250 m*M* imidazole. During the first dialysis (50 m*M* Tris–HCl pH 8.0, 500 m*M* NaCl, 10 m*M* β-mercaptoethanol), the recombinant proteins were treated with thrombin for cleavage of the octahistidine tag. The final purification step consisted of gel filtration of the protein extracts on a Superdex 200 FPLC column equilibrated with 50 m*M* Tris pH 8.0, 500 m*M* NaCl, 10 m*M* β-mercaptoethanol. Protein fractions were pooled, dialyzed into 12.5 m*M* Tris pH 8.0, 125 m*M* NaCl, 5 m*M* DTT, concentrated to at least 10 mg ml^−1^ and finally stored at −80°C. SDS–PAGE analysis was used to confirm the protein purity. Protein concentrations were determined using the absorbance at 280 nm.

A codon-optimized synthetic gene for benzophenone synthase from *Hypericum androsaemum* (HaBPS), GenBank accession AAL79808, as reported by Liu *et al.* (2003 ▸) was purchased from GenScript (Piscataway, New Jersey, USA). The initial HaBPS construct (pUC57 vector) was digested with NcoI and BamHI, gel-purified and ligated with NcoI/BamHI-digested pHIS8. The correct reading frame of the HaBPS pHIS8 construct was achieved using QuikChange PCR and the primers HaBPS-forward, 5′-CTGGTTCCGCGTGGTTCC**ATG**GCCAATTC-3′, and HaBPS-reverse, 5′-GAATTGGCC**ATG**GAACCACGCGGAACCAG-3′ (NcoI sites are underlined and the translation start sites are shown in bold). Primers were purchased from Integrated DNA Technologies (USA) and the sequence of the final pHIS8 construct was confirmed by sequencing (Eton Biosciences, USA).

### 2.3. qPCR analysis of *MdCHS2*, *MdBIS1* and *MdBIS3* gene expression   

Tissues from *M. domestica* ‘Royal Gala’ were collected and qPCR analyses of gene expression were performed as described previously (Henry-Kirk *et al.*, 2012 ▸; Dare *et al.*, 2013 ▸). In brief, all reactions were performed using the LightCycler 480 SYBR Green I Master Mix (Roche Diagnostics) according to the procedure described by the manufacturer. qPCR reactions were performed four times using 5 µl SYBR Green Master Mix, 0.5 µl forward primer, 0.5 µl reverse primer, 1 µl water and 3 µl cDNA template (Table 1 ▸). A negative water control was included in each run. Fluorescence was measured at the end of each annealing step. Amplification was followed by a melting-curve analysis with continual fluorescence data acquisition during the 65–95°C melt. Actin from *M. domestica* (GenBank accession CN938023) was selected as the reference gene because of its consistent transcription level throughout fruit tissues and leaves. The results were interpreted using the LightCycler480 software platform.

### 2.4. Crystallization of MdCHS2, MdBIS3 and HaBPS   

Crystals of MdCHS2, MdBIS3 and HaBPS were grown by the hanging-drop vapor-diffusion method using buffered protein solutions (10–15 mg ml^−1^) mixed with equal volumes of the reservoir solution and incubated at 4°C. Typical reservoir solutions were 14% PEG 8000, 0.3 *M* ammonium acetate, 0.1 *M* sodium MOPSO pH 7.0 for MdCHS2 and 14% PEG 8000, 0.3 *M* ammonium acetate, 0.1 *M* sodium HEPES pH 7.5 for MdBIS3. Crystals of HaBPS grew from reservoirs consisting of 18%(*w*/*v*) PEG 8000, 0.1 *M* PIPES buffer pH 6.5. Crystal-soaking experiments were conducted by transferring apo MdBIS3 crystals into fresh drops containing reservoir plus 15 m*M* benzoyl-CoA and incubating overnight at 4°C. MdCHS2 and MdBIS3 crystals grew within 48 h, while HaBPS crystals typically grew within one week.

### 2.5. X-ray diffraction data collection   

Crystals were transferred to a cryoprotectant solution consisting of reservoir solution supplemented with 20%(*v*/*v*) racemic 1,3-butanediol, prior to immersion in liquid nitrogen. X-ray diffraction data were measured from cooled crystals using ADSC Quantum 315 CCD detectors on beamlines 8.2.1 and 8.2.2 of the Advanced Light Source, Lawrence Berkeley National Laboratory. Diffraction intensities were indexed and integrated with *MOSFLM* (Leslie, 2006 ▸) and *SCALA* (Evans, 2006 ▸) in the *CCP*4 software suite (Collaborative Computational Project, Number 4, 1994 ▸; Potterton *et al.*, 2003 ▸; Winn *et al.*, 2011 ▸).

### 2.6. X-ray structure determination of MdCHS2, MdBIS3 and HaBPS   

Initial crystallographic phases for apo crystals were determined by molecular replacement with *Phaser* (McCoy *et al.*, 2007 ▸). Homology models of MdCHS2 and MdBIS3 were constructed with *MODELLER* (Šali & Blundell, 1993 ▸) based on the structure of chalcone synthase from *Medicago sativa* (PDB entry 1bi5; Ferrer *et al.*, 1999 ▸). A homology model of HaBPS was generated using the crystallographic model of MdBIS3 as a template. These homology models served as the starting models for molecular replacement. The *PHENIX* suite of programs (Adams *et al.*, 2010 ▸) was used to rebuild these initial structural models and for subsequent structural refinements. *Coot* (Emsley & Cowtan, 2004 ▸) was used for graphical map inspection and manual rebuilding of atomic models. Programs from the *CCP*4 suite were employed for all other crystallographic calculations. Molecular visualizations were generated with *PyMOL* (Schrödinger).

### 2.7. *In vitro* enzyme assays   

Steady-state kinetic analyses were performed for chalcone synthase (EC 2.3.1.74), benzophenone synthase (EC 2.3.1.220) and biphenyl synthase (EC 2.3.1.177 and EC 2.3.1.208) activities. Standard enzyme assays for MdCHS2 consisted of 0.1 *M* potassium phosphate buffer pH 7.0, 0.5 µ*M* protein, 60 µ*M* coumaroyl-CoA and 100 µ*M* malonyl-CoA in a total volume of 500 µl. Standard enzyme assays for MdBIS1 and MdBIS3 consisted of 0.1 *M* potassium phosphate buffer pH 7.0, 0.1 µ*M* protein, 15 µ*M* starter substrate, 60 µ*M* malonyl-CoA in a total volume of 500 µl. Assays for HaBPS consisted of 0.1 *M* potassium phosphate buffer pH 7.0, 0.1 µ*M* protein, 20 µ*M* starter substrate and 80 µ*M* malonyl-CoA in a total volume of 500 µl. The reactions were initiated by the addition of protein and were quenched with acetic acid [5%(*v*/*v*)] after 10 min incubations at 37°C, except for MdCHS2, which was incubated for 5 min at 37°C. Kinetic constants were determined using six substrate concentrations with the concentration of the second substrate held constant. Product formation for each protein was linear with respect to incubation time and protein concentration. After quenching, samples were extracted with ethyl acetate, dried under vacuum and then resuspended in 40 µl methanol. Each substrate–enzyme series was assayed in triplicate, and kinetic constants were calculated using a product standard curve and nonlinear regression analyses in *GraphPad Prism* (GraphPad Software).

Product formation was analyzed by LC-MS with an Agilent 1100 series LC-MSD instrument containing a Gemini (4.6 × 150 mm, 5 µm particle size) reversed-phase column. Chromatographic separations employed a flow rate of 0.5 ml min^−1^ and a linear gradient with the initial and final mobile phases consisting of 95%(*v*/*v*) water, 5%(*v*/*v*) acetonitrile, 0.1%(*v*/*v*) formic acid and 5%(*v*/*v*) water, 95%(*v*/*v*) acetonitrile, 0.1%(*v*/*v*) formic acid, respectively. The detection wavelengths were 228 nm (3,5-dihydroxybiphenyl), 288 nm (4-hydroxy­coumarin and naringenin) and 306 nm (phlorobenzo­phenone). Products were confirmed by mass determination and reference to the chromatographic elution of authentic standards.

### 2.8. Molecular phylogenetics   

Amino-acid sequences were aligned using the *PROMALS*3*D* web server (Pei *et al.*, 2008 ▸) and phylogenetic analysis used the Jones–Taylor–Thornton substitution model (Jones *et al.*, 1992 ▸) within the *MEGA*7 software package (Kumar *et al.*, 2016 ▸).

### 2.9. Data deposition   

The cDNA sequences have been deposited in GenBank with accession numbers JQ582624 for MdCHS2, JQ582625 for MdBIS1 and JQ582626 for MdBIS3. Crystallographic models have been deposited in the PDB with the accession codes listed in Table 2 ▸.

## 3. Results   

### 3.1. Functional characterization of CHS, BIS and BPS   

Initially, we isolated three cDNAs from an apple cDNA library (*M. domestica* ‘Royal Gala’) that showed similarity to plant type III PKSs. *BLAST* searches and analysis of the apple genome showed that one of the cDNAs was >96% identical in amino-acid sequence to chalcone synthases from *Malus* (Dare *et al.*, 2013 ▸). The other two cDNAs were 92–99% identical in amino-acid sequence to biphenyl synthases from *Sorbus aucuparia* and *M. domestica* ‘Holsteiner Cox’ (Liu *et al.*, 2007 ▸, 2010 ▸; Chizzali *et al.*, 2011 ▸). Based on sequence similarities, functional studies and phylogenetic analyses (see below), we named the above genes identified in this study *MdCHS2*, *MdBIS1.1* and *MdBIS3.1*. For the sake of brevity, *MdBIS1.1* and *MdBIS3.1* will be referred to as *MdBIS1* and *MdBIS3* in this manuscript.

*In vitro* assays confirmed the biochemical activity of MdCHS2 as a chalcone synthase and of MdBIS1 and MdBIS3 as biphenyl synthases (Table 2 ▸). MdCHS2 was active with 4-coumaroyl-CoA; however, neither biphenyl or benzo­phenone products (see below) could be detected when benzoyl-CoA was used as the starter substrate. Both MdBIS1 and MdBIS3 were active with benzoyl-CoA and salicoyl-CoA, producing 3,5-dihydroxybiphenyl and 4-hydroxycoumarin, respectively. We did not detect significant differences between MdBIS1 and MdBIS3 in their catalytic efficiencies for benzoyl-CoA compared with salicoyl-CoA. In contrast to the observations by Chizzali *et al.* (2011 ▸) that the MdBIS1 homolog in ‘Holsteiner Cox’ occurred as a nonfunctional allele, our biochemical analyses showed that MdBIS1 from ‘Royal Gala’ is a functional enzyme.

**Figure 2:**
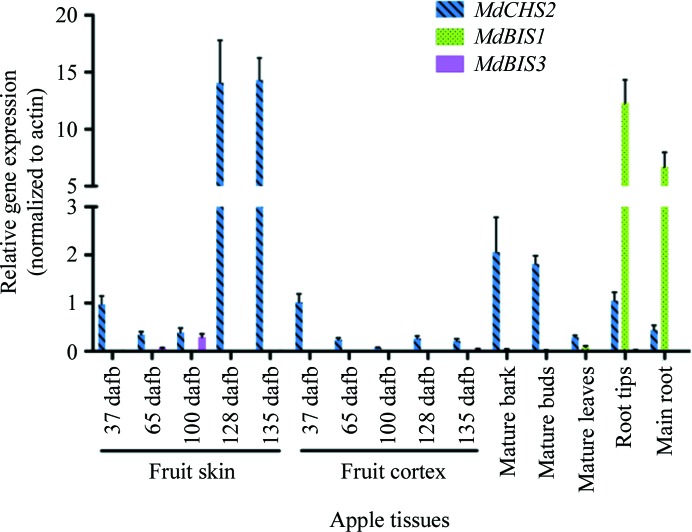
qPCR analysis of *MdCHS2*, *MdBIS1* and *MdBIS3* gene-expression patterns in various tissues of *M. domestica* ‘Royal Gala’. dafb, days after full bloom.

To verify that the cloned *MdCHS2*, *MdBIS1* and *MdBIS3* genes were expressed, we analysed their gene-expression profiles in apple tissues (Fig. 2 ▸). Transcripts of *MdBIS1* were unexpectedly observed to be most abundant in the root tips and main roots of apples and were nearly absent in all other tissues. The *MdBIS1* transcript accumulation in root tips was approximately equal to the accumulation of *MdCHS2* transcripts in apple fruit skin 128 d after full bloom. CHS significantly contributes to the pigmentation of apple fruit skin, which reaches a maximum at approximately 128 d after full bloom in Royal Gala (Henry-Kirk *et al.*, 2012 ▸). Transcript accumulation of *MdBIS3* was similar to *MdCHS2* in apple fruit skin 100 d after full bloom.

For comparative purposes, we obtained a synthetic gene derived from the gene sequence of *H. androsaemum* BPS (HaBPS). Similar to previous reports, HaBPS produced 2,4,6-trihydroxybenzophenone (phlorobenzophenone) when incubated with benzoyl-CoA (Table 2 ▸; Liu *et al.*, 2003 ▸). Biphenyl products were not detected. Additionally, unlike MdBIS1 and MdBIS3, 4-hydroxycoumarin was not detected when HaBPS was incubated with salicoyl-CoA as a starter substrate.

### 3.2. Overall structures of MdCHS2, MdBIS3 and HaBPS crystals   

**Figure 3:**
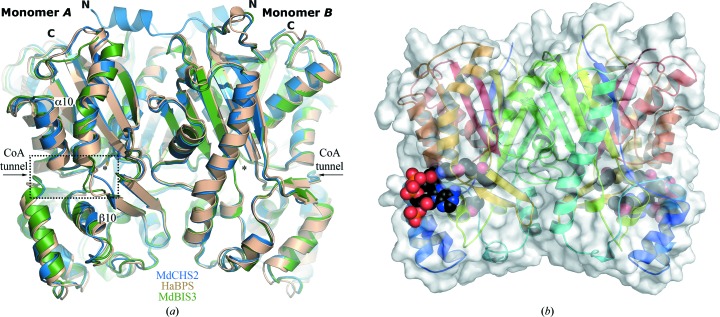
Overall crystal structures of MdCHS2, MdBIS3 and HaBPS. (*a*) The asymmetric units of MdCHS2 (blue), MdBIS3 (green) and HaBPS (brown) contain the physiologically relevant homodimers. The polypeptide chains of each protein are represented as ribbons. The side chains of the active-site cysteines for each crystal structure are shown as sticks and labeled with asterisks. The N-terminus and C-terminus of the superposed structures are labeled N and C, respectively. The dashed box highlights the CoA tunnel and the displaced solvent loops near the CoA tunnels of BIS and BPS (see Fig. 4 ▸). (*b*) The structure of BIS complexed with benzoyl-CoA. The surface of the protein dimer is displayed and each monomeric subunit is shown in ribbon representation with rainbow coloring from the N-terminus (blue) to the C-terminus (red). Benzoyl-CoA is displayed as spheres with atoms color-coded according to element: carbon, black; oxygen, red; phosphorus, orange; nitrogen, blue; sulfur, yellow.

The crystal structures of MdCHS2, HaBPS and MdBIS3 were solved by molecular replacement (Table 3 ▸). All crystals contained the physiologically relevant twofold rotationally symmetric homodimer in their asymmetric unit (Fig. 3 ▸). The crystallographic model of MdCHS2 contained 774 out of 778 residues. The crystallographic models of HaBPS and MdBIS3 contained 772 out of 790 residues and 757 out of 776 residues, respectively. Lastly, the crystallo­graphic model of MdBIS3 complexed with benzoyl-CoA contained 756 out of 780 residues. The missing residues of all crystallo­graphic models were at the N- and C-termini of each monomeric subunit. Additionally, each monomer of MdBIS3 contained electron density consistent with 1,3-butanediol, the cryoprotectant. When superposed, the polypeptide backbones of MdCHS2 and MdBIS3 have an r.m.s.d. of 0.9 Å, those of MdCHS2 and HaBPS have an r.m.s.d. of 0.7 Å and those of MdBIS3 and HaBPS have an r.m.s.d. of 0.9 Å. Our final crystallographic models of MdCHS2 and HaBPS were refined at 2.1 Å resolution (*R* = 16.6%; *R*_free_ = 20.9%) and 2.85 Å (*R* = 20.1%; *R*_free_ = 26.1%), respectively. The final crystallographic models of apo MdBIS3 and MdBIS3 complexed with benzoyl-CoA were refined at 1.17 Å (*R* = 10.6%; *R*_free_ = 12.6%) and 1.20 Å (*R* = 11.1%; *R*_free_ = 13.1%), respectively, the highest resolution structures obtained to date for a type III PKS.

Crystallographic analysis of secondary-structural elements between MdCHS2, HaBPS and MdBIS3 revealed only minor differences in topology, none of which immediately explained the differences in substrate specificity and terminal cyclization preferences during catalysis. The five-layered αβαβα topology of secondary-structural elements of each monomer is consistent with the ‘thiolase fold’ originally observed in thiolase and present in all known structures of type III PKSs (Austin & Noel, 2003 ▸; Mathieu *et al.*, 1994 ▸). Each monomer of MdCHS2, MdBIS3 and HaBPS contains two *cis*-peptide bonds at the dimer interfaces which are well defined by the electron densities of each structure and are conserved in all known type III PKSs. Structural differences between the superposed C^α^ traces of MdCHS2, HaBPS and MdBIS3 occur primarily at the solvent interface. In particular, a three-residue solvent-exposed loop in MdBIS3 and HaBPS is displaced approximately 5 Å toward the CoA tunnel compared with MdCHS2 (Fig. 3 ▸ *a*).

#### 3.2.1. CoA tunnel   

**Figure 4:**
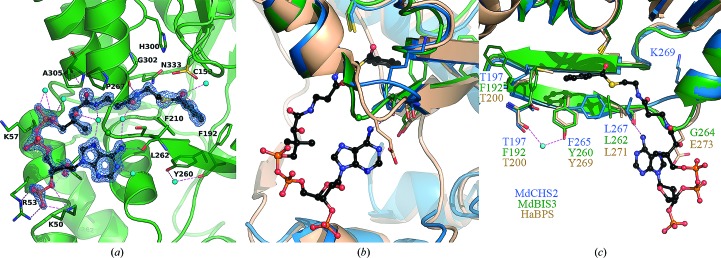
Binding interactions of benzoyl-CoA. (*a*) Co-crystallization of MdBIS3 with benzoyl-CoA. MdBIS3 is displayed as a green cartoon; benzoyl-CoA is displayed in ball-and-stick representation. Hydrogen bonds are shown as dashed lines and waters are shown as spheres. Atoms are color-coded according to element: carbon, black; oxygen, red; nitrogen, blue; sulfur, yellow. The 2*F*_o_ − *F*_c_ OMIT electron-density difference map surrounding the benzoyl-CoA ligand is displayed as blue-colored cages and contoured at σ = 1.0 with carve = 1.3. (*b*) Overlay of the MdBIS3–benzoyl-CoA complex with MdCHS2 and HaBPS. Residues associated with the displaced solvent-exposed loop near the CoA tunnel and mutations near the benzoyl moiety of benzoyl-CoA are displayed as sticks. (*c*) Overlay of the MdBIS3–benzoyl-CoA complex with MdCHS2 and HaBPS rotated approximately 120° counterclockwise from that shown in (*b*).

A conserved arrangement of hydrogen bonds assists in the binding of CoA substrates in the CoA tunnels of MdBIS3, HaBPS and MdCHS2. Despite the use of reducing agents in all buffers and crystallization solutions, the electron-density maps indicated that the active-site cysteines of the MdBIS3 crystals were oxidized to their sulfinic acid derivatives (–SO_2_H). Oxidation of active-site cysteines has been observed in other type III PKS structures (PDB entries 1bi5 and 1qlv) and prevents the transfer of substrates onto the active-site cysteines (Jez, Austin *et al.*, 2000 ▸; Ferrer *et al.*, 1999 ▸). Nonetheless, insight into substrate binding was provided by co-crystallization of MdBIS3 with benzoyl-CoA, resulting in intact benzoyl-CoA molecules with partial occupancies in both monomers of MdBIS3 (Figs. 3 ▸ *b* and 4 ▸ *a*). In MdBIS3, the side chains of residues Lys50 and Arg53 as well as the backbones of Ala305 and Gly302 form hydrogen bonds to the phosphates and pantetheine subunits of benzoyl-CoA, respectively. These hydrogen bonds are conserved in other type III PKS structures containing bound CoA substrates (PDB entries 1bq6 and 1ee0; Ferrer *et al.*, 1999 ▸; Jez, Austin *et al.*, 2000 ▸). However, as mentioned above, both MdBIS3 and HaBPS contain a three-residue solvent-exposed loop that is displaced significantly toward the CoA tunnel (Fig. 4 ▸ *b*). The displaced loop in the MdBIS3–benzoyl-CoA complex generates an additional hydrogen bond between the adenine unit of benzoyl-CoA and the backbone carbonyl of Leu262. The displaced loops found in MdBIS3 and HaBPS appear to correlate with the rotameric state of one of the gatekeeper residues (Fig. 4 ▸ *c*). The gatekeeper residues, Phe215 and Phe265 in MdCHS2, separate the CoA tunnel from the internal active-site cavity and affect substrate selectivity by acting as a steric gate during substrate binding (Ferrer *et al.*, 1999 ▸; Jez *et al.*, 2002 ▸). Phe265 of CHS is replaced with tyrosine in both MdBIS3 (Tyr260) and HaBPS (Tyr269). Differences in the side-chain torsion angles of Tyr260 in MdBIS3 (χ_1_ = 61°) and Tyr269 in HaBPS (χ_1_ = 50°) compared with Phe265 of CHS (χ_1_ = 170°) significantly reorient the tyrosine side chains and increase the conformational flexibility of the nearby Leu262 in MdBIS3 and Leu271 in HaBPS. Lastly, the side-chain conformation of Tyr260 in MdBIS3 is stabilized *via* the presence of a water-mediated hydrogen bond to the backbone carbonyl of Phe192. The F265Y mutation in CHS was one of three mutations needed to shift the substrate specificity of CHS towards benzoyl-CoA (Liu *et al.*, 2003 ▸). Presumably, a similar water-mediated hydrogen bond stabilizes Tyr269 of HaBPS; however, the lower resolution of the diffraction data for the HaBPS crystals prevented the analysis of waters.

### 3.3. Active-site architectures of CHS, BIS and BPS   

#### 3.3.1. Substrate loading   

**Figure 5:**
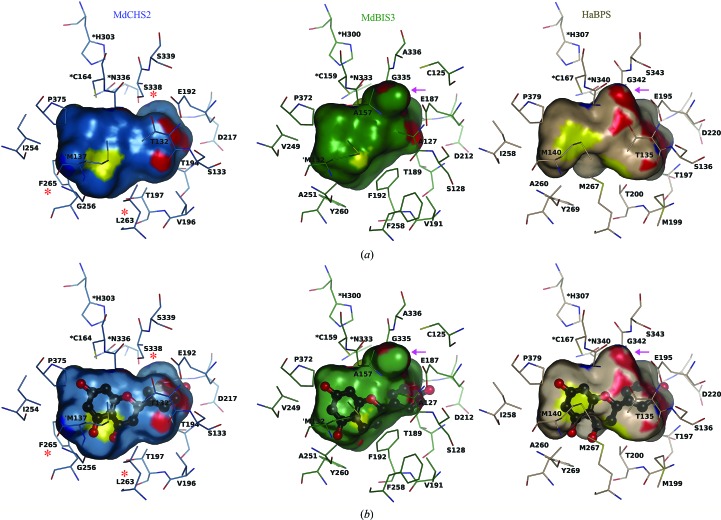
Active-site architectures of MdCHS2, MdBIS3 and HaBPS. (*a*) The internal cavities of MdCHS2 (blue), MdBIS3 (green) and HaBPS (brown) are displayed as surface representations. Residues lining the cavities are displayed; the catalytic triads of each protein are labeled with black asterisks. Red asterisks highlight residues in CHS that are known to affect benzoyl-CoA substrate specificity. The novel pockets of MdBIS3 and HaBPS are labeled with magenta arrows. Residues are colored according to element: oxygen, red; nitrogen, blue; sulfur, yellow. (*b*) Naringenin, the product of CHS activity with 4-coumaroyl-CoA as a substrate, is modeled into the cavities of MdCHS2, HaBPS and MdBIS3. Naringenin is displayed in ball-and-stick representation. Coordinates for naringenin were obtained from superpositioning of the CHS–naringenin complex (PDB entry 1cgk). C atoms of the naringenin ligand are colored black and all other elements are colored as described above.

The active-site cavity of MdCHS2 is conserved and isomorphous with the previously characterized cavity of *M. sativa* CHS (Ferrer *et al.*, 1999 ▸). In contrast, the active-site cavities of MdBIS3 and HaBPS contain a series of hydrophobic residues that block access to the 4-coumaroyl-CoA binding region of CHS. Residues of the MdCHS2 pocket include Ser133, Glu192, Thr194, Thr197, Gly216 and Ser338. Ser133, Glu192 and Thr914 are conserved in MdBIS3 as Ser128, Glu187 and Thr189, while Thr197, Gly216 and Ser338 are replaced with Phe192, Ala211 and Gly335. The conspicuous replacement of Thr197 in MdCHS2 with Phe192 in MdBIS3 reshapes the bottom of the MdBIS3 cavity (Fig. 5 ▸ *a*). Furthermore, Phe192 sterically clashes with the carbonyl of naringenin derived from coumaroyl-CoA when the previously characterized CHS–naringenin complex is superposed onto MdBIS3 (Fig. 5 ▸ *b*; Ferrer *et al.*, 1999 ▸). Although Thr197 of MdCHS2 is conserved in HaBPS (Thr200), the electron density of Thr200 in HaBPS supports a rotamer with the methyl group of threonine protruding up into the coumaroyl-binding pocket of CHS (Fig. 5 ▸ *a*). A significant change in the HaBPS cavity arises from the small-to-large replacement of Leu263 in MdCHS2 with Met267 in HaBPS. The side chain of Met267 in HaBPS is in the same vicinity of the active-site cavity as the Phe192 side chain of BIS, and similarly clashes with the carbonyl unit of naringenin derived from coumaroyl-CoA in the CHS–naringenin complex (Fig. 5 ▸ *b*). Met267 of HaBPS is replaced with Phe258 in MdBIS3. The orientation of the side chain of Phe258 away from the binding pocket suggest that it is not directly involved in binding benzoyl-CoA, although it may assist in stabilizing the conformation of Phe192 in MdBIS3. Lastly, Gly256 is on the surface of the MdCHS2 cavity near the CoA tunnel; in MdBIS3 and HaBPS this position contains the slightly bulkier Ala. Mutations that increase the bulkiness of the side chain of position 256, including G256A, have previously been shown to shift the substrate specificity of CHS towards smaller substrates (Jez, Austin *et al.*, 2000 ▸). Collectively, hydrophobic mutations in the active sites of MdBIS3 and HaBPS provide hydrophobic surfaces for interacting with small hydrophobic substrates such as benzoyl-CoA.

#### 3.3.2. Elongation and cyclization within benzoic acid-specific type III PKSs   

The replacement of Ser338 in MdCHS2 with Gly generates a novel pocket in the active-site cavities of MdBIS3 and HaBPS associated with their elongation and cyclization reactions. Ser338 of MdCHS2 resides at the intersection of the coumaroyl-binding pocket and elongation/cyclization cavity and stabilizes the conformation of elongated polyketide intermediates (Jez, Austin *et al.*, 2000 ▸). The replace­ment of Ser338 in CHS with Gly in both MdBIS3 (Gly335) and HaBPS (Gly342) allows the internal cavities of MdBIS3 and HaBPS to expand behind their respective active-site cysteines to generate novel pockets (Fig. 5 ▸ *a*). The sides of this novel pocket in MdBIS3 are formed from the side chains of Cys125, Ala127, Ala157, Glu187 and Ala336 as well as the main-chain atoms of Gly158, Ala157, Gly335 and Ala336. Similarly, the side chains of Thr135 and Glu195 and the main-chain atoms of Gly166, Gly342 and Ser343 line the sides of this novel pocket in HaBPS. Notably, Klundt *et al.* (2009 ▸) observed that mutagenesis of Thr135 in HaBPS disrupted chain elongation and cyclization specificity.

**Figure 6:**
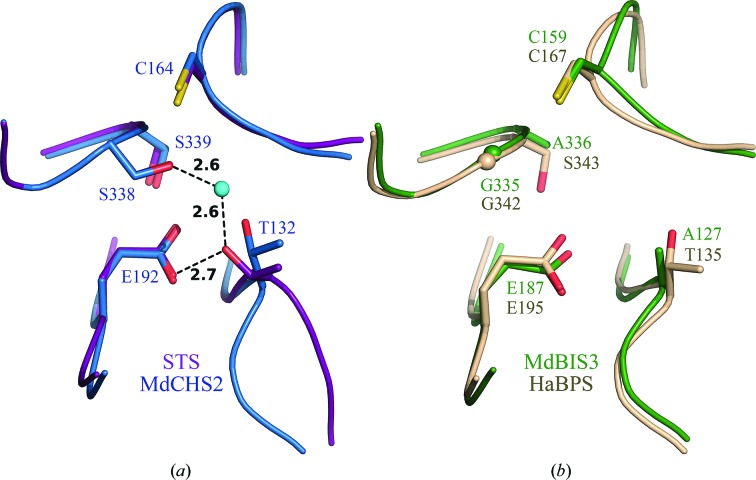
Structural elements contributing to cyclization specificity in MdCHS2, MdBIS3 and HaBPS. (*a*) The aldol-switch residues of stilbene synthase (PDB entry 1u0u) superposed onto MdCHS2 (blue). Residues are displayed as sticks. Atoms are color-coded according to element: oxygen, red; nitrogen, blue; sulfur, yellow. Water is shown as a cyan sphere. Hydrogen bonds are shown as dashed lines with associated distances in Å. (*b*) Active-site regions of MdBIS3 and HaBPS, homologous to the stilbene aldol switch, are overlayed and displayed as sticks.

The structural determinants of cyclization specificity in benzoic acid-specific type III PKSs are distinct from the previously described ‘aldol switch’ of STSs. As mentioned above, CHS and BPS catalyze terminal Claisen cyclizations of their polyketide intermediates, while STS and BIS catalyze terminal aldol cyclizations (Fig. 1 ▸). Previously, the emergence of a subtle water-mediated hydrogen-bond network involving the side chains of Thr132, Glu192 and Ser338 (CHS numbering) was found to underlie aldol-cyclization reactions in STSs (Austin, Bowman *et al.*, 2004 ▸; Shomura *et al.*, 2005 ▸; Fig. 6 ▸ *a*). The aldol switch of STSs is disrupted in BIS by the replacement of Thr132 and Ser338 of MdCHS2 with Ala (Ala127) and Gly (Gly335), respectively (Fig. 6 ▸ *b*). Both Ala127 and Gly335 line the sides of the novel pocket formed in the active-site cavity of MdBIS3. While there are ordered waters present in the novel pocket of MdBIS3 which could function similarly to the nucleophilic water of the STS aldol switch, the lower resolution diffraction data of HaBPS prohibited further structural comparisons.

## 4. Discussion   

The displaced solvent-exposed loop of MdBIS3 and HaBPS has also been noted in the structures of 2-pyrone synthase and stilbene synthase (Jez, Austin *et al.*, 2000 ▸; Austin, Bowman *et al.*, 2004 ▸). In the CoA complexes of MdBIS3 and 2-pyrone synthase, their displaced loops provide an additional hydrogen bond and van der Waals contacts to CoA ligands. The displaced loops of MdBIS3 and HaBPS correspond to the region B loop of pine STS described by Austin *et al.* (2004 ▸). Mutagenesis of residues in the pine STS region B loop by Austin *et al.* (2004 ▸) did not alter the cyclization specificity of pine STS. Interestingly, the region B loop in grapevine STS contains a Pro residue (Pro269) that exhibits a strong positive-selection pattern in a d*N*/d*S* analysis; an indicator that this residue and loop played a significant role in the emergence of the large stilbene synthase gene family in grapevine (Parage *et al.*, 2012 ▸). Pro269 of grapevine STS is not conserved in MdBIS3 or HaBPS and there does not appear to be a consensus sequence for the displaced loop. Nonetheless, displacement of the region B loop is not observed in any available CHS structures. Furthermore, structural comparisons indicate that the rotameric state of the gatekeeper residue Phe265 (CHS numbering) consistently differs between CHS and functionally divergent type III PKSs (non-CHS). Thus, it appears that rotameric differences of Phe265 and the associated displacement of the three-residue solvent-exposed loop are likely to affect the kinetics and/or thermodynamics of acyl-CoA binding during type III PKS catalysis.

Benzoic acid-specific type III PKSs contain mutations that reshape their active-site cavities to favor the binding of small hydrophobic substrates. Replacement of Thr197 in CHS with Phe in MdBIS3 (Phe192) appears to be responsible for closing off access to the coumaroyl-binding region of CHS. Similarly, the side chain of Met267 of HaBPS replaces Leu263 of CHS and blocks access to the coumaroyl-binding area of the CHS cavity. Phe192 in MdBIS3 and Met267 in HaBPS also provide hydrophobic surfaces that would favorably interact with small hydrophobic substrates and their polyketide intermediates. Met267 of HaBPS is substituted with a Phe in MdBIS3 (Phe258) and seems to primarily function in stabilizing the conformation of nearby residues including Phe192. A triple mutant of CHS, L263M/F265Y/S338G, preferred benzoyl-CoA over 4-coumaroyl-CoA as a substrate (Liu *et al.*, 2003 ▸). The mutations of Phe265 in CHS to Tyr and of Ser338 to Gly are present in all known benzoic acid-specific type III PKSs. Conversely, the mutations of Thr197 in CHS to Phe and of Leu263 in CHS to Met appear to be specific to BPS and BIS, respectively (Supplementary Fig. S1).

A novel pocket in the active-site cavity of MdBIS3 and HaBPS, in a region known to affect elongation and cyclization, arises from the replacement of Ser338 of CHS with Gly. The side chains of Ala127 in MdBIS3 and Thr135 in HaBPS line the sides of the novel pocket. Mutagenesis of Thr135 in HaBPS to Leu resulted in a reduction in chain-elongation steps from three to two and a shift in the terminal cyclization mechanism from Claisen to lactonization (Klundt *et al.*, 2009 ▸). As a result of changes in the number of chain-elongation steps and cyclization specificity, the T135L mutant of HaBPS produced phenylpyrone (6-phenyl-4-hydroxy-2*H*-pyran-2-one) as the dominant product. Interestingly, homology modeling of HaBPS by Klundt *et al.* (2009 ▸) suggested that the mutation T135L generated a novel pocket that sterically favored the generation of phenylpyrone over phlorobenzophenone. Further structural and functional analyses are needed to deconvolute steric *versus* electronic effects on catalysis within this novel pocket of benzoic acid-specific type III PKS.

The structural basis of the terminal aldol cyclization of BIS is different from the aldol switch of STSs, yet the chemical mechanisms are likely to be similar. The replacement of Thr132 and Ser338 of STS with Ala127 and Gly335 prohibits the water-mediated hydrogen-bond networks underlying the terminal aldol cyclizations of STSs from forming in BIS. Sequence differences between the residues lining the walls of the novel pockets in BPS (T_135_, G_342_SAC) and BIS (A_127_, G_335_APT/S) are a useful starting point for further exploration of cyclization specificity using mutagenesis. Additionally, the novel pocket of MdBIS3 contains ordered waters that could function similar to the nucleophilic water that is essential for the aldol switch of stilbene synthases. However, higher resolution structures of BPS are needed for comparisons of subtle changes in backbone, side chain and ordered waters between BPS and BIS. Interestingly, benzal­acetone synthase, another type III PKS that contains mutations that prohibit the formation of the STS aldol switch, relies on a nucleophilic water for the terminal aldol-like decarboxylation of its diketide intermediate (Morita *et al.*, 2010 ▸). Although the specific nucleophilic waters of STSs and benzalacetone synthase are not present in our MdBIS3 structures, we speculate that a nucleophilic water in the novel pocket of BIS is a critical element for the structure-based cyclization mechanism of BISs.

**Figure 7:**
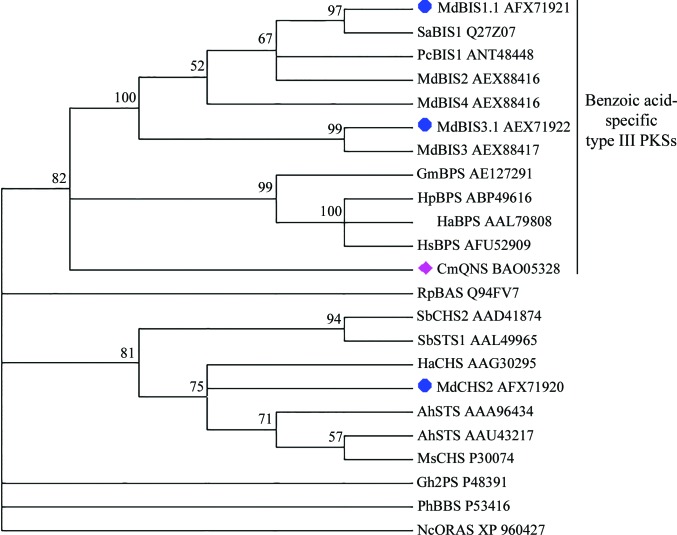
Maximum-likelihood phylogenetic tree showing the relationship of CmQNS to MdBIS3, HaBPS and other plant type III PKSs. Blue circles indicate the sequences described in this report; a magenta diamond indicates the CmQNS sequence. Bootstrap values for 500 replicates are shown above the branches. Branches with a bootstrap value of less than 50% were collapsed. GenBank accession numbers are given at the end of the gene names. Abbreviations used are as follows: BIS, biphenyl synthase; QNS, quinolone synthase; BPS, benzophenone synthase; BAS, benzalacetone synthase; CHS, chalcone synthase; STS, stilbene synthase; 2PC, 2-pyrone synthase; BBS, bibenzyl synthase; ORAS, 3′-oxoresorcinolic acid synthase.

**Figure 8:**
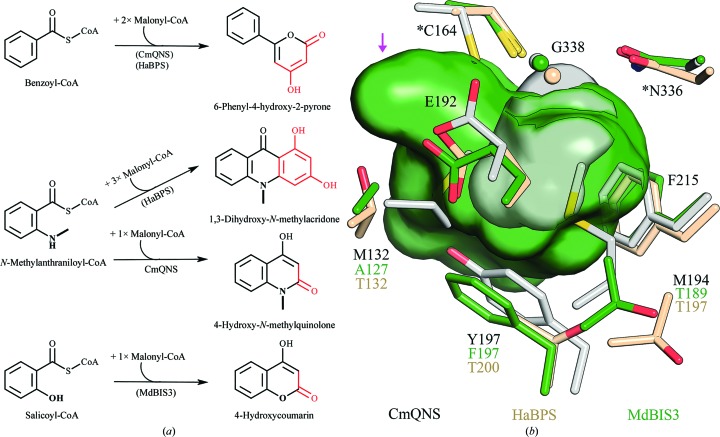
Comparison of the active sites and reactions of CmQNS, MdBIS3 and HaBPS. (*a*) Reactions catalyzed by CmQNS, HaBPS and MdBIS3. Proteins in parentheses indicate that displayed reactions are promiscuous activities. (*b*) Surface representations of MdBIS3 and CmQNS. Residues lining the internal cavities of CmQNS (grey), MdBIS3 (green) and HaBPS (brown) are displayed as sticks. Atoms are color-coded according to element: oxygen, red; nitrogen, blue; sulfur, yellow. Conserved residues have only a single label. Residues forming part of the catalytic triad are denoted with asterisks. The magenta arrow identifies the novel pocket in the MdBIS3 and HaBPS cavities. Coordinates for CmQNS were obtained from PDB entry 3wd8.

The functional and structural diversity of benzoic acid-specific type III PKSs is likely to be broader than those of BIS and BPS. Unexpectedly, in our survey of the literature we found a quinolone synthase from *Citrus microcarpa* (CmQNS) which grouped with BIS and BPS in a molecular phylogenetic analysis (Mori *et al.*, 2013 ▸). Our independent analysis of MdBIS3 and HaBPS phylogeny support the observations of Mori *et al.* (2013 ▸) that CmQNS is closely related to BIS and BPS (Fig. 7 ▸). CmQNS produces 4-hydroxy-*N*-methylquinolone from the decarboxylative condensation of *N*-methylanthraniloyl-CoA with malonyl-CoA followed by lactonization of the diketide intermediate (Fig. 8 ▸ *a*). Significantly, Mori *et al.* (2013 ▸) observed that CmQNS had significant promiscuous activity with benzoyl-CoA, yielding phenylpyrone as a single product. Phenylpyrone is a minor side product of wild-type BPS activity with benzoyl-CoA; however, as described above, a single point mutation shifts the activity of BPS to produce phenylpyrone as the dominant product (Klundt *et al.*, 2009 ▸). Moreover, BPS also has promiscuous activity with *N*-methyl­anthraniloyl-CoA, yielding the acridone product after three chain-elongation steps (Liu *et al.*, 2003 ▸). Although BIS has not been reported to turn over *N*-methylanthraniloyl-CoA, the promiscuous activity of BIS with salicoyl-CoA is similar to the CmQNS reaction (Fig. 8 ▸ *a*). Firstly, *N*-methylanthraniloyl-CoA and salicoyl-CoA contain structurally similar substitutions of the phenyl ring at the *ortho* position. Secondly, CmQNS and BIS activity with salicoyl-CoA involves a single chain-elongation step before lactonization of their respective diketide intermediates. Consequently, the quinolone products of CmQNS and 4-hydroxycoumarin, the final product of BIS activity with salicoyl-CoA, are structurally similar. Notably, the catalytic efficiencies of BIS with salicoyl-CoA and benzoyl-CoA are similar (Table 2 ▸). Unfortunately, kinetic parameters for CmQNS activity with benzoyl-CoA have not been reported.

The active-site cavity of CmQNS has several structural features similar to those of BIS and BPS. Firstly, the CmQNS structure (PDB entry 3wd8) contains several mutations consistent with BIS and BPS, namely the replacement of Thr197, Gly256, Leu263 and Ser338 in CHSs with Tyr197, Ala256, Met263 and Gly338 (Mori *et al.*, 2013 ▸). Secondly, these mutations eliminate the coumaroyl-binding pocket of CHSs in a similar fashion as in MdBIS3 and HaBPS (Fig. 8 ▸ *b*). Lastly, CmQNS contains a displaced solvent-exposed loop near the CoA tunnel comparable to MdBIS3 and HaBPS. Nevertheless, the active-site cavity of CmQNS does not include the novel pocket found in BIS and BPS. The novel active-site pocket of BIS and BPS is blocked in CmQNS because of a series of methionine substitutions at residues 132 and 194. Additionally, significant changes in the backbone positions of the active-site cysteine and nearby residues help to block formation of the novel BIS/BPS pocket (Fig. 8 ▸ *b*). Collectively, the functional, structural and phylogenetic data discussed above lead us to hypothesize that CmQNS is a descendent of a benzoic acid-specific type III PKS recruited into alkaloid metabolism.

## 5. Conclusions   

In conclusion, we report here the first structural analyses of benzoic acid-specific type III PKSs. Benzoic acid-specific type III PKSs catalyse the committed steps in the biosynthesis of benzophenone and biphenyl metabolites in plants. The products of BIS and BPS, 3,5-dihydroxybiphenyl and phloro­benzophenone, respectively, are subsequently modified *via* an assortment of tailoring reactions (for example, hydroxylation, *O*-methylation and prenylation) to yield the final repertoire of biphenyl, dibenzofuran and phloro­benzophenone metabolites found in plants (Khalil *et al.*, 2015 ▸; El-Awaad *et al.*, 2016 ▸; Sircar *et al.*, 2015 ▸; Fiesel *et al.*, 2015 ▸). BIS and BPS contain several small-to-large mutations which block the binding of 4-coumaroyl-CoA and provide hydrophobic surfaces for smaller hydrophobic starter CoAs and intermediates. Significantly, BIS and BPS contain a novel pocket in their active-site cavities associated with their chain-elongation and cyclization reactions. These structural idiosyncrasies underlie the preference of BIS and BPS for benzoic acid-derived substrates. Furthermore, the promiscuous nature of BIS and BPS may have contributed to the emergence of a type III PKS involved in alkaloid biosynthesis. The renowned promiscuity of type III PKSs is likely to underpin their ability to evolve as their host organisms interacted with constantly changing environments (Weng & Noel, 2012 ▸). The structural snapshots presented in this paper deepen our understanding of structure–function relationships in the type III PKS family and lay a foundation for ongoing efforts to exploit the biosynthetic potential of plant metabolic enzymes.

## Supplementary Material

PDB reference: benzophenone synthase, 5uco

PDB reference: chalcone synthase, 5uc5

PDB reference: biphenyl synthase, 5w8q

PDB reference: biphenyl synthase, complex with benzoyl-CoA, 5wc4

Supplementary Figure S1: multiple sequence alignment.. DOI: 10.1107/S2059798317016618/ag5010sup1.pdf

## Figures and Tables

**Table 1:** Primers for expression analysis by qPCR

Gene	Direction	Sequence (nt)
*MdActin*	Forward	AGTAATTTCCTTGCTCATTCGGTCA
	Reverse	GATGTGGATTGCCAAAGCTGAGTA
*MdCHS2*	Forward	CAGCGTTGATTTATCTATCTGCTTCTGC
	Reverse	TCCACCAAGTTAACCCCATGACG
*MdBIS1*	Forward	GCGCCTTTGGTTAAGAATCATGGA
	Reverse	TCTCCTCTGTTAGATGCAAGTAACGTTTTC
*MdBIS3*	Forward	ACAGCCGTGCTGCGTAGTGAATC
	Reverse	TGCAGCAGGGGTGTCTATACACTGG

**Table 2:** Apparent steady-state kinetic parameters for MdCHS2, MdBIS1, MdBIS3 and HaBPS *k*_cat_ and *K*_m_ are expressed as the mean (± standard error) of triplicate measurements.

	4-Coumaroyl-CoA			Benzoyl-CoA			Salicoyl-CoA			Malonyl-CoA
Protein	*k*_cat_ (min^−1^)	*K*_m_ (µ*M*)	*k*_cat_/*K*_m_ (*M* ^−1^ s^−1^)	*k*_cat_ (min^−1^)	*K*_m_ (µ*M*)	*k*_cat_/*K*_m_ (*M* ^−1^ s^−1^)	*k*_cat_ (min^−1^)	*K*_m_ (µ*M*)	*k*_cat_/*K*_m_ (*M* ^−1^ s^−1^)	*K*_m_ (µ*M*)
MdCHS2	1.2 ± 0.1	17.9 ± 6.1	1117	—	—	—	—	—	—	12.8 ± 2.7
MdBIS1	—	—	—	17.0 ± 0.1	0.8 ± 0.2	3.5 × 10^5^	12.5 ± 0.1	1.6 ± 0.4	1.3 × 10^5^	6.8 ± 1.7
MdBIS3	—	—	—	8.1 ± 0.0	0.5 ± 0.1	2.7 × 10^5^	6.0 ± 0.1	1.1 ± 0.4	0.9 × 10^5^	4.1 ± 1.9
HaBPS	—	—	—	1.1 ± 0.0	1.3 ± 0.5	1.4 × 10^4^	—	—	—	2.8 ± 0.7

**Table 3:** Summary of X-ray diffraction data-collection and refinement statistics Values in parentheses are for the highest resolution shell.

	MdCHS2	MdBIS3	MdBIS3–benzoyl-CoA	HaBPS
Resolution range (Å)	40.99–2.10 (2.18–2.10)	27.92–1.17 (1.21–1.17)	50–1.20 (1.22–1.20)	46.24–2.85 (2.95–2.85)
Space group	*P*2_1_2_1_2	*P*2_1_	*P*2_1_	*C*222_1_
*a*, *b*, *c* (Å)	118.8, 56.6, 111.5	55.3, 113.0, 62.8	55.2, 113.0, 62.8	82.9, 121.8, 185.0
α, β, γ (°)	90, 90, 90	90, 93.1, 90	90, 93.3, 90	90, 90, 90
Monomers per asymmetric unit	2	2	2	2
Total reflections	201461	1126833	876363	1187706
Multiplicity	4.8 (4.8)	4.5 (2.5)	3.7 (3.4)	3.5 (2.4)
Completeness (%)	94.4 (90.3)	98.2 (93.2)	99.7 (99.6)	85.0 (85.0)
Mean *I*/σ(*I*)	10.5 (3.8)	11.3 (3.0)	12.2 (3.4)	4.5 (2.2)
Wilson *B* factor (Å^2^)	32.6	7.4	8.8	24.3
*R*_merge_	0.092 (0.496)	0.084 (0.297)	0.069 (0.477)	0.253 (0.498)
*R*_work_	0.159 (0.207)	0.106 (0.166)	0.111 (0.173)	0.201 (0.239)
*R*_free_	0.212 (0.270)	0.126 (0.190)	0.131 (0.191)	0.261 (0.335)
No. of non-H atoms				
Total	6364	7222	7230	5793
Macromolecules	6018	6039	6065	5748
Ligands	0	12	124	0
Water	346	1171	1041	45
No. of protein residues	780	757	756	754
R.m.s.d., bonds (Å)	0.005	0.012	0.015	0.004
R.m.s.d., angles (°)	0.92	1.49	1.66	0.69
Ramachandran favored (%)	98	98	98	96
Ramachandran outliers (%)	0	0	0	0.1
Clashscore	0.66	0.34	2.21	0.70
Average *B* factor (Å^2^)				
Overall	38.10	11.80	13.70	13.70
Macromolecules	37.90	9.90	11.30	13.70
Ligands		14.50	16.10	
Solvent	41.10	22.10	27.70	10.00
PDB code	5uc5	5w8q	5wc4	5uco
