# Supplementary material for: Molecular architectures of benzoic acid-specific type III polyketide synthases
Source: Acta Crystallogr D Struct Biol. 2017 Nov 30;73(Pt 12):1007–19. doi: 10.1107/S2059798317016618 (PMC5713876; doi:10.1107/S2059798317016618)
Supplement: Supplementary file 1 [file d-73-01007-sup1.pdf]

STRUCTURAL BIOLOGY

Volume 73 (2017)

Supporting information for article:

Molecular architectures of benzoic acid-specific type III polyketide synthases

Charles Stewart, Kate Woods, Greg Macias, Andrew Allan, Roger Hellens and Joseph Noel

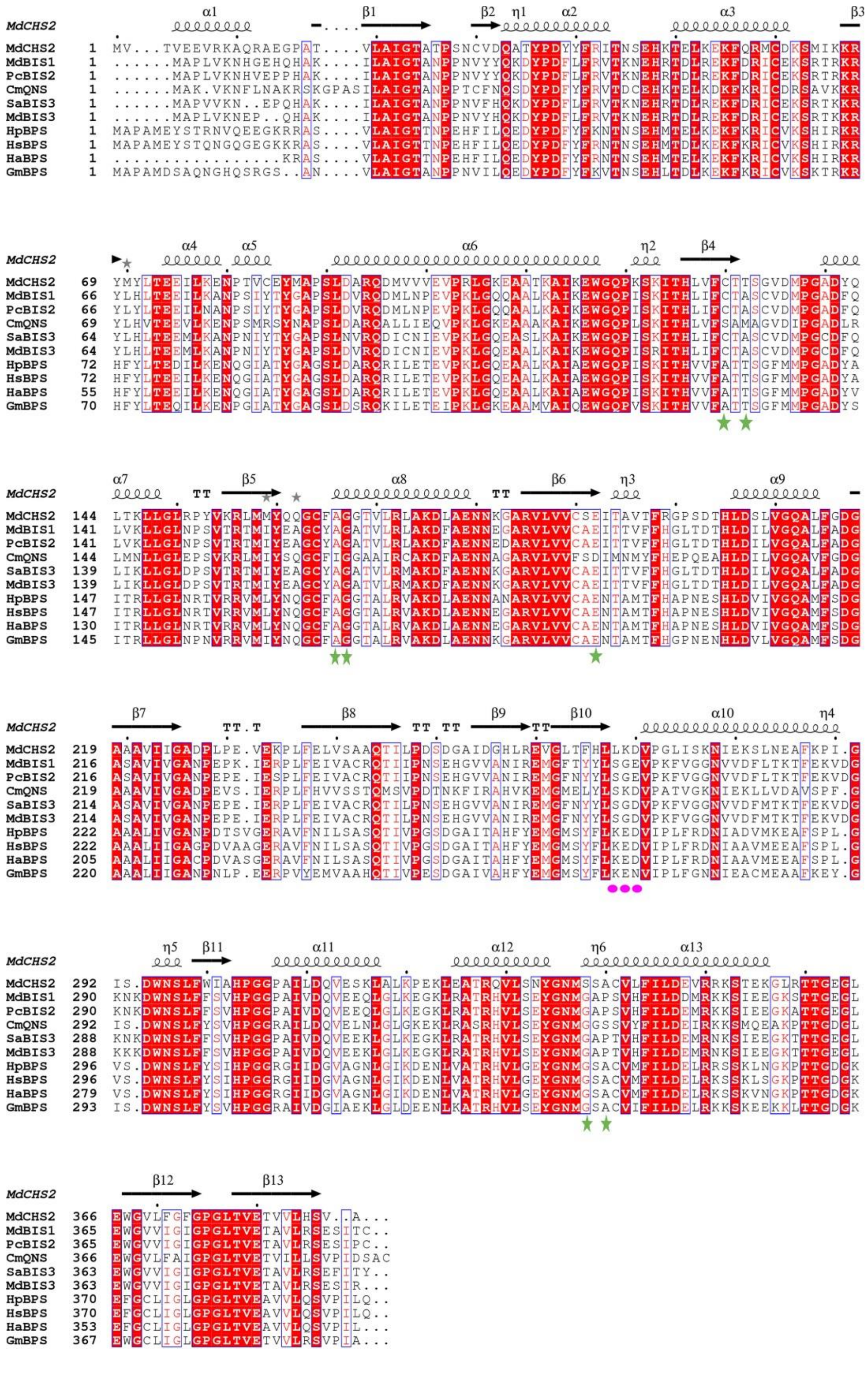

Figure S1 Multiple sequence alignment of benzoic acid-specific type III PKSs with type III PKS homologs. Strictly conserved residues are highlighted in red with white lettering. Columns containing at least 80% identical residues are boxed in blue with red lettering for conserved residues. Magenta ovals indicate solvent displaced loops in MdBIS3 and HaBPS. Green stars indicate residues lining novel pocket within MdBIS3 and HaBPS.
